# Decoding *Arabidopsis thaliana* CPK/SnRK Superfamily Kinase Client Signaling Networks Using Peptide Library and Mass Spectrometry

**DOI:** 10.3390/plants13111481

**Published:** 2024-05-27

**Authors:** Nagib Ahsan, Amr R. A. Kataya, R. Shyama Prasad Rao, Kirby N. Swatek, Rashaun S. Wilson, Louis J. Meyer, Alejandro Tovar-Mendez, Severin Stevenson, Justyna Maszkowska, Grazyna Dobrowolska, Qiuming Yao, Dong Xu, Jay J. Thelen

**Affiliations:** 1 Division of Biochemistry, Christopher S. Bond Life Sciences Center, University of Missouri, Columbia, MO 65211, USA; 2 Department of Chemistry and Biochemistry, Mass Spectrometry, Proteomics and Metabolomics Core Facility, Stephenson Life Sciences Research Center, The University of Oklahoma, Norman, OK 73019, USA; 3 Center for Bioinformatics, NITTE Deemed to be University, Mangaluru 575018, India; 4 Medical Research Council Protein Phosphorylation and Ubiquitylation Unit, School of Life Sciences, University of Dundee, Dundee DD1 5EH, UK; 5 Arvinas, Inc., New Haven, CT 06511, USA; 6 Bayer Crop Science, St. Louis, MO 63141, USA; 7 Elemental Enzymes, St. Louis, MO 63132, USA; 8 Institute of Biochemistry and Biophysics, Polish Academy of Sciences, ul. Pawińskiego 5a, 02-106 Warsaw, Polanddobrowol@ibb.waw.pl (G.D.); 9 Department of Electrical Engineering & Computer Science, Christopher S. Bond Life Sciences Center, University of Missouri, Columbia, MO 65211, USA

**Keywords:** kinase client assay, kinase substrates, mass spectrometry, phosphorylation, signaling network

## Abstract

Members of the calcium-dependent protein kinase (CDPK/CPK) and SNF-related protein kinase (SnRK) superfamilies are commonly found in plants and some protists. Our knowledge of client specificity of the members of this superfamily is fragmentary. As this family is represented by over 30 members in *Arabidopsis thaliana*, the identification of kinase-specific and overlapping client relationships is crucial to our understanding the nuances of this large family of kinases as directed towards signal transduction pathways. Herein, we used the kinase client (KiC) assay—a relative, quantitative, high-throughput mass spectrometry-based in vitro phosphorylation assay—to identify and characterize potential CPK/SnRK targets of Arabidopsis. Eight CPKs (1, 3, 6, 8, 17, 24, 28, and 32), four SnRKs (subclass 1 and 2), and PPCK1 and PPCK2 were screened against a synthetic peptide library that contains 2095 peptides and 2661 known phosphorylation sites. A total of 625 in vitro phosphorylation sites corresponding to 203 non-redundant proteins were identified. The most promiscuous kinase, CPK17, had 105 candidate target proteins, many of which had already been discovered. Sequence analysis of the identified phosphopeptides revealed four motifs: LxRxxS, RxxSxxR, RxxS, and LxxxxS, that were significantly enriched among CPK/SnRK clients. The results provide insight into both CPK- and SnRK-specific and overlapping signaling network architectures and recapitulate many known in vivo relationships validating this large-scale approach towards discovering kinase targets.

## 1. Introduction

The ability of cells to respond to both developmental and environmental cues depends on complex intracellular signaling networks [1]. Understanding these signaling networks provides insight into regulatory mechanisms, the coordination of responses to cues, and ultimately, the control of cellular function and fate [2,3]. Many approaches use system-related analyses to decipher the underlying structure of cellular signaling and how changes in signaling affect the flow of information [4,5,6]. One of the essential cellular signaling systems is mediated via Ca^2+^ homeostasis, which is regulated by complex interactions among pumps, channels, exchangers, and Ca^2+^-binding proteins [7,8]. Cytoplasmic calcium (Ca^2+^_cyt_) is a second messenger in plant signaling [9] and essential to many aspects of growth and development, as well as interaction with and response to environmental perturbations [10,11]. Stimulus-characteristic intracellular Ca^2+^ signals are generated by a variety of inputs including changes in temperature [12], light [13], and gravitational vector [14], and interactions with microbes [15], and wounding and herbivory [16]. Changes in cytoplasmic calcium [(Ca^2+^)_cyt_] are then translated into protein phosphorylation and signal transduction [17,18]. Many of the targets responsive to cytoplasmic Ca^2+^ consist of transcription factors, protein kinases, and phosphoprotein phosphatases, which contain Ca^2+^-responsive cis-elements.

Protein phosphorylation is the most widespread post-translational modification and is critical in signal transduction and biochemical regulation [19,20,21]. The *Arabidopsis thaliana* genome encodes at least 1029 protein kinases (PK) [21], many of which are Ca^2+^-dependent or regulated. The nomenclature of Ca^2+^-regulated protein kinases can be confusing, especially if contemplating trans-kingdom comparisons [22,23]. Herein, we have adopted the system described by Hrabak et al. [24], the CDPK-SnRK superfamily (CSS) comprises seven types of plant protein kinases: calcium-dependent protein kinases (CPKs), CPK-related kinases (CRKs), phosphoenolpyruvate carboxylase kinases (PPCKs), PEP carboxylase kinase-related kinases (PEPRKs), calmodulin-dependent protein kinases (CaMKs), calcium and CaM-dependent protein kinases (CCaMKs), and SNF1-related kinases (SnRKs). A consolidating factor of many members of the CSS is that they are able to perceive changes in (Ca^2+^)cyt, which allows them to function as Ca^2+^-signal transducers [24,25,26]. Ca^2+^ perception may occur either directly via binding of Ca^2+^ to intrinsic EF-hands, or indirectly via CSS binding to Ca^2+^-responsive trans elements. In the case of CDPKs, Ca^2+^-regulation occurs primarily via direct binding of Ca^2+^ to C-terminal EF-hands, with limited examples of additional indirect modulation through binding to Ca^2+^-responsive proteins such as CaMs [27]; while, in the case of SnRK3s, or CBL-Interacting Protein Kinases (CIPKs) as they are otherwise known, Ca^2+^ activation occurs indirectly via binding to calcineurin B-like (CBL) calcium-binding proteins [28].

Much of our current understanding of phospho-regulation in plant signaling pathways is the result of *A. thaliana* mutants [29,30,31,32] and phosphoproteomic analyses [33,34,35]. While there are a few examples of kinases dedicated to a single client protein [36,37], a comparison of the number of *A. thaliana* protein kinase genes [38,39] coupled with the number of experimentally mapped phosphorylation sites (PhosPhAt, http://phosphat.uni-hohenheim.de/; P3DB, http://p3db.org/) indicates that a multiple client repertoire is likely the norm for most kinases [35,40]. Both the act of discovering kinase clients and of defining kinase client networks, however, are technically challenging. Synthetic peptides have often been employed for the in vitro determination of kinase clients. In the 1970s, E.G. Krebs’ lab pioneered the use of a synthetic 7-mer peptide (Kemptide) based upon the phosphorylation site of pig liver pyruvate kinase as a PKA client [41]. A decade later, Syntide, a synthetic 18-mer based on phosphorylation site 2 of mammalian glycogen synthase, was introduced as a CDPK client [42]. Subsequently, synthetic peptides in a broad range of presentations have been used for both in vitro kinase characterization [29,43,44] and for the in vivo discovery of kinase clients [45,46]. Other studies, summarized in [47], used surface-based assays for identifying in vitro client proteins from human, yeast, and plant kinases [48,49,50]. Such surface-based assays suffer from artifacts due to non-native conformations of surface-bound proteins, and due to molecular crowding effects [47].

Leveraging the fact that kinase-substrate specificities are largely determined by the primary amino acid sequence of phosphorylation motifs, we developed a quantitative synthetic peptide-based mass spectrometry assay, the kinase client assay (KiC assay), for client discovery and network analysis [37,47,51,52]. The KiC assay consolidates a label-free synthetic peptide kinase assay with the use of LC-MS/MS to identify kinase–client relationships, map phosphorylation sites, and to quantify kinase activity and specificity. The quantitative analysis of phosphorylation-site specificity was essential to define signaling network topology and PK–client relationships [53]. In that regard, kinase activity can be determined using the KiC assay by monitoring the spectral counts of phosphorylated and unphosphorylated peptides [47,51]. Overall, the information obtained from the use of the KiC assay has contributed to our understanding of diverse kinases and signaling pathways [6,52,54,55,56,57,58,59,60,61].

Proof-of-concept studies validated the use of the KiC assay for the analysis of multisite phosphorylation [37,47,52]. Here, we employed the quantitative, label-free, MS-based KiC assay to identify new CSS–client relationships from a library of 2095 synthetic peptides representing experimentally identified phosphorylation sites. CSS kinase–client relationships and phosphorylation sites were identified and combined with results from co-expression analyses to forge a kinase client network. Pinpointing these previously unknown CSS clients and their specific phosphorylation sites will help us understand the CSS and Ca^2+^ signaling networks.

## 2. Materials and Methods

### 2.1. Synthesis and Development of the 2.1K Peptide Library

Based on the previously established in vivo phosphoproteomic dataset for *A. thaliana* available in P^3^DB [35], a library comprising 2095 synthetic 10- to 20-mer peptides was designed (Table S1) and prepared by PEPscreen (Sigma, St. Louis, MO, USA). This 2.1k synthetic peptide library is the basis of our integrated experimental strategy for the identification of kinase–client relationships. Stock solutions were prepared by dissolving peptides in 80% (*v*/*v*) dimethylformamide in water to an approximate final concentration of 8 mM, due to variations in synthesis yields. These synthetic peptides have diverse physiochemical and biological properties. For instance, the hydrophobicity of these peptides [62] ranged from −6 to 75 (Figure S1A). However, the hydrophobicity of more than 65% of the peptides was within the range of 15 to 35. The more positive values indicate a higher hydrophobicity. Furthermore, 70% of these peptides are Ser-phosphorylated residues, 22% are Thr-phosphorylated, and 8% are Tyr-phosphorylated (Figure S1B). Cognate proteins representing the peptide sequences have diverse subcellular localizations, with 44% targeted to nucleus (Figure S1C). In an effort to homogenize the wide range of hydrophobicity (Figure S1A), ten pools were generated, each with a uniform distribution of hydrophobic peptides (Figure S1D). Each pool contains an approximate equimolar distribution of between 208 and 211 peptides. Samples from the stock solutions were then diluted into the KiC assay [47,52].

### 2.2. Preparation of Recombinant Protein Kinases

Recombinant protein kinases were prepared as described previously [52]. Briefly, total RNA was isolated from *A. thaliana* ecotype Col-0 plants grown under standard glasshouse long-day (16 h) conditions (22 °C, 50% relative humidity, 144 μmol photons m^−2^ s^−1^) using the RNeasy Mini Kit (Qiagen, Valencia, CA, USA). The cDNAs were synthesized using M-MLV reverse transcriptase (Promega, Madison, WI, USA). The coding regions for each protein kinase (PK) were amplified using Pfu polymerase (Stratagene, Santa Clara, CA, USA) with PK-specific primers (Table S2). Purified PCR products were directionally cloned into the Champion pET200 TOPO vector (Invitrogen, Carlsbad, CA, USA) according to the manufacturer’s protocol. All constructs were sequenced to verify that no changes had been introduced during amplification.

*Escherichia coli* BL21 Star (DE3) was transformed by heat shock. After the induction of heterologous protein expression by the addition of IPTG to 0.5–1.0 mM, cells were grown with continuous shaking (200 rpm) for 4 h at 37 °C or overnight at 18 °C, depending upon the PK. Cells were harvested by centrifugation at 6000× *g* for 15 min. Cell pellets were suspended in 50 mM NaH_2_PO_4_, pH 8.0, containing 300 mM NaCl and 10 mM imidazole, and then broken by three passages through a French pressure cell at 12,000 psi. Cell debris was removed by centrifugation at 10,000× *g* for 15 min at 4 °C. Supernatants were loaded onto Ni-NTA affinity columns (Qiagen, Valencia, CA, USA), and, after washing, bound proteins were eluted with 250 mM imidazole-HCl, pH 8.0, containing 50 mM NaH_2_PO_4_, and 300 mM NaCl. Eluted His_6_-proteins were dialyzed overnight to remove the imidazole and then stored in 10 mM Tris-HCl, pH 7.5, containing 1 mM DTT, and 50% (*v*/*v*) glycerol at −20 °C until used.

The recombinant AtSnRK proteins were purified precisely as previously described [63,64]. After purification, glycerol was added to a final concentration of 10% (*v*/*v*), and the kinases were stored at −80 °C until used.

### 2.3. Purity and Activity Assessment of Recombinant Kinases

The preliminary purity of the recombinant proteins/kinases was evaluated by SDS–PAGE. However, each kinase activity of was further demonstrated by individual in vitro kinase assays using the commercially available synthetic peptide Syn-tide 2 (Sigma-Aldrich, St. Louis, MO, USA) followed by mass spectrometry.

### 2.4. The Kinase Client Assay

The KiC assay was performed according to our previously published protocol [47,52]. Briefly, the conditions for the in vitro KiC assay (Figure S2) include the kinase buffer (20 mM HEPES-KOH, pH 7.4, 5 mM MgCl_2_, 1 mM DTT, and 2 mM ATP), the purified recombinant PKs, and pools of synthetic peptides [47,52]. The detailed diagrammatic representation of the KiC assay is shown in Figure S2. When assaying CPKs, reactions additionally contained 0.2 mM CaCl_2_. For each kinase, separate reactions were performed with each of the peptide pools (Pool 1–10; Figure S1D).

### 2.5. LC-MS/MS Analysis

The freeze-dried peptides were dissolved by adding 40 µL of 0.1% (*v*/*v*) formic acid (FA) for MS analysis. Samples were loaded into 96-well plates, and then were placed onto a pre-chilled 10 °C auto-sampler. Ten μL of each sample was analyzed using a Finnigan Surveyor liquid chromatography (LC) system attached on an LTQ Orbitrap XL ETD mass spectrometer (Thermo Fisher, San Jose, CA, USA). During LC, peptides were bound to a C8 Captrap (Michrom Bioresources, Auburn, CA, USA), eluted with an acetonitrile (ACN) gradient, and then separated using a “Magic C18” (200 Å, 5 μ bead, Michrom Bioresources) fused silica column (10 cm × 150 μm, Polymicro Technologies, Phoenix, AZ, USA). Each column was washed with a gradient of 95% to 5% (*v*/*v*) ACN in 0.1% (*v*/*v*) FA before sample analysis.

Analysis of synthetic peptides was performed using LTQ Orbitrap XL ETD. The peptides were fragmented using either collision-induced dissociation (CID) or by using a “decision tree” method that utilizes both CID and ETD during a single sample analysis [65]. Instrument conditions were exactly as previously described [52]. Briefly, nano-spray ionization source parameter settings were as follows: ion spray voltage (kV), 2.10; capillary temperature (°C), 250; capillary voltage (v), 36; and tube lens (v), 90. Precursor masses were scanned with the analyzer set to FTMS mass range, normal; resolution, 60,000 or 100,000; scan type, positive mode; data type, centroid; and a scan range of 200–2000 *m*/*z*. The ten most abundant ions from the precursor scan were selected for subsequent fragmentation using the ion trap analyzer, at a normal mass range, normal scan rate, and centroid data type. Charge-state screening and mono-isotopic precursor-selection modes were enabled. Unassigned charge states and masses with a charge state of +1 were not analyzed. The CID data-dependent scan settings were collision energy 35 kV, default charge state +2, isolation width 2.0 *m*/*z*, an activation time of 30 milliseconds (ms), and multistage activation was disabled. Dynamic exclusion was enabled with a repeat count of three, repeat duration of 30 ms, exclusion list size 50–100, and exclusion duration of 30 ms. Data-dependent ions fragmented with ETD had an exclusion mass width of 10 ppm. The reagent ion source settings including temperature, emission current, energy level, and CI pressure were 160 °C, 50 μA, −70 V, and 17.5 psi, respectively. The activation time was 100 ms, and supplemental activation mode was enabled.

### 2.6. Database Search

For the analysis of the results of the KiC assay, raw MS files were searched against a decoy database consisting of a random complement of sequences comprising the peptide library, using SEQUEST (Proteome Discoverer, v. 1.0.3, Thermo Fisher). The instrument and detailed search parameters were described previously [52]. Identification data were evaluated using the XCorr function of SEQUEST, and phosphorylation-site localization was accomplished using phosphoRS (Proteome Discoverer, v. 1.0.3, Thermo Fisher). The XCorr values for each charge state were set to default, and no decoy hits were allowed. For final validation, each spectrum was inspected manually and accepted only when the phosphopeptide had the highest pRS-site probability, pRS score, XCorr value, and the site-determining fragment ions allowed the unambiguous localization of the phosphorylation site.

### 2.7. Motif Analysis

Motifs were extracted using the motif-x [66] web server (http://motif-x.med.harvard.edu/; accessed on 14 April 2024). Pre-aligned foreground (central character S or T, 308 Ser, and 65 Thr phosphorylation sites for CDPKs) and background (5921 Ser and 2086 Thr sites from 2095 synthetic peptides) sequences (13-mers) were analyzed using motif-x [66,67]. The minimum occurrence threshold was 5 and the significance value was less than 0.00003 (*p* << 0.01 after Bonferroni correction). Results are displayed using the WebLogo [68].

### 2.8. Identification of Kinase Client Network

A CDPK clients network was constructed using Cytoscape 3.2 [69]. The CDPK client protein–protein interaction, co-expression, and subcellular localization information were explored as some of the network parameters. The experimentally known and predicted high confidence protein–protein interaction data were collected from various sources, such as TAIR (http://www.arabidopsis.org/; accessed on 14 April 2024), AtPID (http://www.megabionet.org/atpid/; accessed on 14 April 2024), AtPIN (http://bioinfo.esalq.usp.br/atpin/; accessed on 14 April 2024), CCSB (http://interactome.dfci.harvard.edu/; accessed on 14 April 2024), PAIR (http://www.cls.zju.edu.cn/pair/; accessed on 14 April 2024), Arabidopsis Interactions Viewer (http://bar.utoronto.ca/interactions/; accessed on 14 April 2024), and STRING9.05 (http://string-db.org/; accessed on 14 April 2024). The co-expression between pairs of genes or proteins was based on the Pearson correlation [70] of log2 transformed normalized expression values from GSE3011 (27 arrays, leaf/inflorescence tissue) for *A. thaliana* present in the NCBI gene expression omnibus (http://www.ncbi.nlm.nih.gov/geo/; accessed on 14 April 2024). The subcellular localization information was collected from TAIR and SUBA3 (http://suba.plantenergy.uwa.edu.au/; accessed on 14 April 2024).

## 3. Results

### 3.1. Mass Spectrometry-Based Kinase Activity Confirmation of the Recombinant Kinases

**Figure 1:**
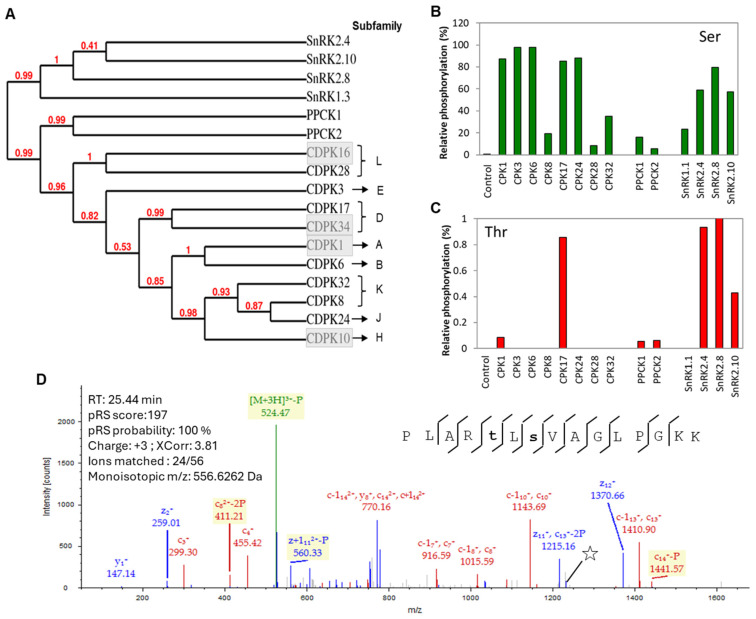
The phylogenetic relationship and the recombinant kinases activity of the CSS kinases used in this study. (**A**) The phylogenetic tree of the CSS kinases used in the current study and by other in vitro studies for the identification of potential clients. The white CPKs indicate at least 2 other in vitro studies used the same CPK, whereas the shaded CPKs represent the same subfamily of the CPKs. (**B**,**C**) Evaluation of CSS kinases by in vitro phosphorylation with the Syntide-2 peptide followed by identification of the phosphorylation sites by MS/MS analysis. Bar diagrams show the relative phosphorylation activity of p-Ser (**B**) and p-Thr (**C**) sites by each kinase. Relative kinase phosphorylation was determined using the total number of peptide spectral matches (PSMs), which is considered as 100% and phosphorylated PSMs. (**D**) The lower panel is a MS/MS spectrum of the Syntide-2 peptide phosphorylated by CPK17. The bold lower-case letters in the inset sequence and the star in the spectrum indicate the sites of phosphorylation and detection of the phosphorylated residue, respectively.

Recombinant proteins were prepared from *A. thaliana* CSS kinases (Figure 1A): CPK1, CPK3, CPK6, CPK8, CPK17, CPK24, CPK28, and CPK32 from the CDPK family; PPCK1 and PPCK2 from the PPCK family; SnRK1.3, SnRK2.4, SnRK2.8, and SnRK2.10 from the SnRK family. All of the recombinant kinases phosphorylated the Ser residue of Syntide-2 synthetic peptide, albeit with differing efficiency (Figure 1B). For instance, CPK1, CPK3, CPK6, CPK17, and CPK24 showed relatively higher phosphorylation of Syntide-2, whereas CPK8, CPK28, CPK32, PPCK1, PPCK2, and SnRK1.3 were comparatively less active. It is noteworthy that CPK17 and the SnRK2 subfamily kinases additionally catalyzed the low-level phosphorylation of the Thr site of Syntide-2 (Figure 1C). Our results suggest that CPK17 is more active than other CPKs because it exhibited higher levels of phosphorylation at both the Ser and Thr sites of the Syntide-2 peptide (Figure 1D). Overall, the quality control experiment verified that the recombinant CCS kinases are active, and the phosphorylation sites are consistent with previous reports of the differential phosphorylation of Syntide-2 by other plant CPKs [29].

### 3.2. Identification of Putative Clients for CDPKs, SnRKs, and PPCKs

The CSS superfamily members such as CDPKs are involved in Ca^2+^ homeostasis and implicated in developmental and stress response, although their client/substrate specificity remains largely unknown. However, a limited number of small- and medium-throughput research studies were implemented to identify the CSS member–client relationships [29,52]. Herein, we prepared a synthetic peptide library composed of 2095 peptides (Table S1, Figure S1). The peptides were extracted from the P^3^DB database and represent 2661 in vivo mapped phosphorylation sites from *A. thaliana*. We then performed the KiC assay [29,52] to identify potential client proteins for members of the *A. thaliana* CSS kinases (Figure S2). The primary evaluation of peptide spectral matches employed the Xcorr scoring function of SEQUEST, which had a 1% false discovery rate when tested against a randomized database derived from the peptide library. Ultimately, phosphorylation sites were localized using the phosphoRS algorithm (Proteome Discoverer, v1.0.3, Thermo Fisher) and phosphopeptides with a pRS score ≥15 and/or a pRS site probability of ≥55% were accepted. The proportion of phosphorylated PSMs against the total number of PSMs identified for a particular site or peptide facilitates the relative quantitation facilitated through the KiC assay.

**Figure 2:**
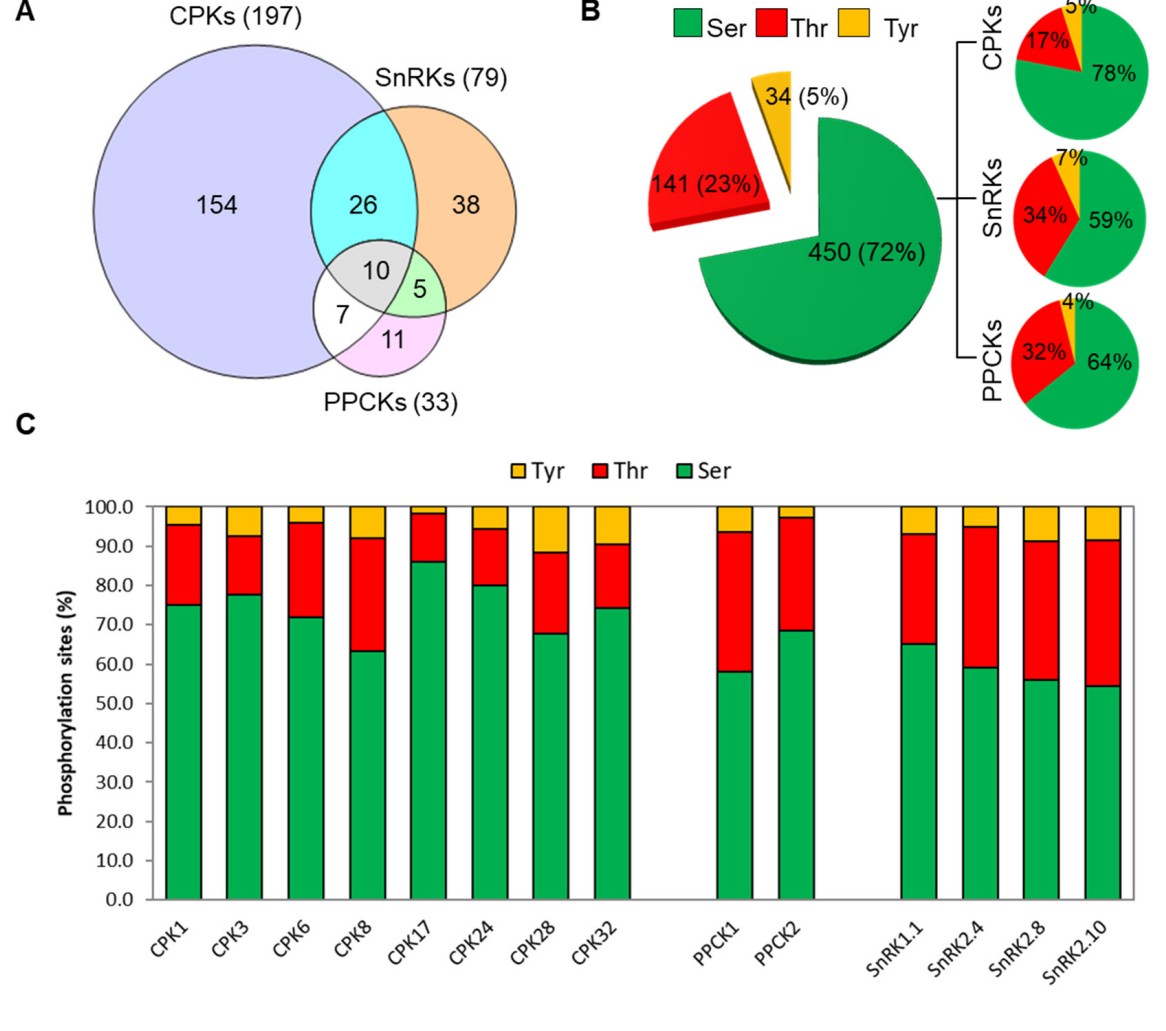
Identification and phosphorylation status of the CSS kinase clients. (**A**) Venn diagram analysis shows the number of unique and overlapped clients identified by CPKs, SnRKs, and PPCKs. The amino acid residues phosphorylated by CPKs, PPCKs, or SnRKs were identified using the synthetic peptide library and the KiC assay. (**B**) The pie chart indicates the overall proportion of Ser, Thr, and Tyr O phosphorylation sites identified. (**C**) The histograms indicate the target residue specificity of individual protein kinases.

Using the recombinant kinases with the 2.1k peptide library in the KiC assay, we were able to identify 197 (Tables S3–S10), 33 (Tables S11 and S12), and 79 (Tables S13–S16) putative client proteins for members of the CDPK, PPCK, and SnRK family kinases, respectively (Figure 2). Detailed descriptions of the kinase clients are presented in Tables S3–S16. In several instances, we observed overlapping client protein identification (Figure 2). A total of 10 clients, including a PAM domain protein (At1g75990), histidine kinase 2 (At5g35750), HAT3.1 (At3g19510), unknown protein (At1g15400), embryonic factor 1 (At2g38280), magnesium chelatase I2 (At5g45930), cell division cycle 48B (At3g53230), aldehyde dehydrogenase 3 (At4g34240), XAP5 circadian timekeeper (At2g21150), and POL-like 5 (At1g07630), were identified as clients for each of the kinases tested (Figure 2A). Overlapping client specificity among the kinase subfamilies was also observed (Figure 2A). For example, a total of 7, 26, and 5 clients were in common between CDPKs and PPCKs, CDPK and SnRKs, and SnRKs and PPCKs, respectively (Figure 2A). Phosphorylation of some clients by multiple members of a kinase family or by members of multiple kinase families is not unusual [29,52]. A total of 154, 11, and 38 non-overlapping clients were identified for the CDPKs, PPCKs, and SnRKs family kinases (Figure 2A).

These putative clients comprised a total of 625 phosphosites, wherein 72%, 23%, and 5% are Ser, Thr, and Tyr phosphosites, respectively (Figure 2B). In case of CDPKs, Ser was the preferred (78%) phosphorylation site, whereas PPCKs and SnRKs showed a higher preference for Thr sites (Figure 2B,C). It is interesting to note that Tyr phosphorylation consisted of 34 sites corresponding to a total of 21 non-overlapping client peptides (Figure 2B). Eight of these peptides were phosphorylated precisely at the same Tyr residue identified by the phosphoproteomics studies conducted by different groups (P^3^DB). For instance, CYP71A16 (At5g42590), and chlorophyll B reduced 2 (At5g18660) were phosphorylated by CPK28. Sucrose-induced receptor kinase 1 (At5g10020) was phosphorylated by CPK32. A “protein kinase family protein” (At3g28690) was phosphorylated by SnRK1.3. Phytanoyl-CoA 2-hydroxylase (At2g01490) and brassinosteroid-signaling kinase 1 (At4g35230) were phosphorylated by SnRK2.4. And, an unknown protein (At1g47900) and B-block binding subunit of TFIIIC (At1g59453) were phosphorylated by SnRK2.8. The relative phosphorylation preference of Tyr residues by the various kinases ranged from 2 to 12% (Figure 2C). For instance, among the CDPK clients, CPK28 and CPK32 clients showed over 10% Tyr phosphorylation, whereas CPK17 showed a much lower preference for Tyr residues. In summary, we were able to identify a vast number of phosphorylation sites and several clients for the tested CSS members.

### 3.3. Reconstruction of In Silico Kinase Client Signaling Networks

**Figure 3:**
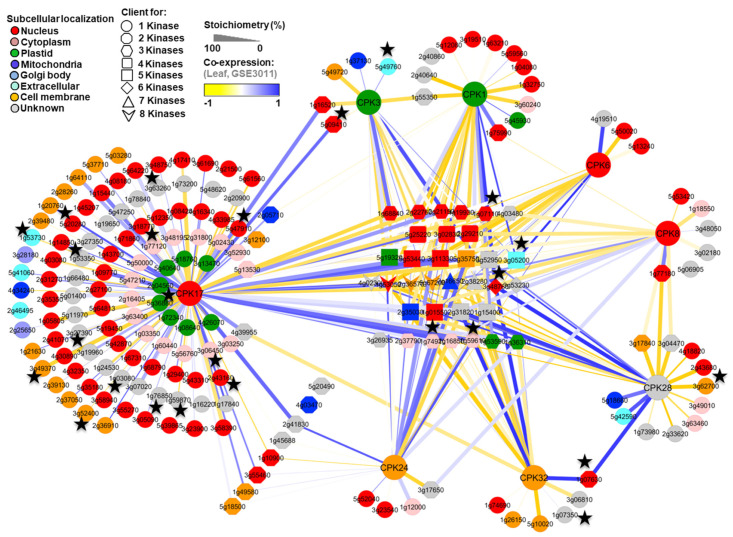
Topological relationships among the CPK cognate client proteins. Data were obtained using recombinant kinases, the KiC assay, and a library containing 2095 synthetic peptides. The cartograph was assembled using Cytoscape 3.0.1 (http://www.cytoscape.org). Nodes of different shapes indicate kinase-specific rather than overlapping clients. Node colors indicate subcellular localization. Edge thickness indicates relative phosphorylation stoichiometry of each site. The co-expression relationships between pairs of genes/proteins is based on the Pearson correlation of log2 transformed normalized expression values from GSE3011 for *A. thaliana* (http://www.ncbi.nlm.nih.gov/geo, accessed on 14 April 2024) and is denoted by edge color. Nodes with a black star are clients that were identified in one or more of the other in vitro studies [29,71,72,73], which were also reported to be P-proteins in vivo, and are phosphorylated at the same amino acid residues (P^3^DB).

To obtain a better understanding of the CPK family client networks, CPK clients were further analyzed by Cytoscape, a network analysis and visualization program. As anticipated, our results showed that, in addition to the kinase-specific clients, several clients were common among the CPKs examined (Figure 3). For instance, a total of four clients: pectin methylesterase 39 (At4g02300), protein kinase superfamily protein (At1g53050), PXY/TDR-correlated 1 (At2g36570), and a leucine-rich repeat protein kinase family protein (At5g67200) were identified as common clients for all eight CPKs (Figure 3). A proteinaceous RNAse P2 (At2g16650), adenosine 5′-monophosphate deaminase (At2g38280), cell division control 2 (At3g48750), and cell division cycle 48B (At3g53230) proteins were common clients across at least six CPKs. Similarly, a total of 10, 3, and 13 clients were common in at least five, four, and three CPKs, respectively (Figure 3). Our results are consistent with the previous study [29], wherein many overlapping clients were also identified among the CPKs, indicating that CPK clients are often shared. Nevertheless, overlapping hits did not represent the majority of CPK clients, indicating a substantial level of isoform specificity as well.

As expected, co-expression, phosphorylation stoichiometry (indicating relative phosphorylation in terms of the phospho and non-phospho peptide spectral match identified for a particular site/peptide), and subcellular localization of these clients showed kinase-specific preferences (Figure 3). For instance, an ethylene-induced calmodulin binding protein (At5g09410) and unknown protein (At1g16520) were identified as common clients for both CPK17 and CPK3; however, phosphorylation stoichiometry and co-expression analyses (Figure 3) showed both of these proteins could be the preferable potential targets for CPK17 rather than CPK3. Similarly, an uncharacterized protein (At2g41830) was identified as common client for both CPK17 and CPK24 and showed high co-expression with both of the kinases; however, the relative phosphorylation stoichiometry was higher for CPK17, suggesting it is a preferred target for that particular CPK.

Similarly, a number of clients were overlapped between PPCK1 and PPCK2 (Figure S3A; Tables S11 and S12). Conversely, there was comparatively little client overlap between SnRK subclasses 1 and 2 (Figure S3B, Tables S13–S16). However, as anticipated, there was more overlap among the SnRK subclass 2 (SnRK 2.4, SnRK 2.8, and SnRK 2.10) clients. 

### 3.4. Cross-Validation of the CPK Clients

To demonstrate the broad applicability of this approach, we next performed a comparison between the KiC assay clients with previously known putative CDPK substrates identified by several in vitro studies [29,71,72,73]. The comparative analysis showed a total of 25 KiC assay clients were in common among the CDPK targets identified in these studies (Figure 3). These overlapped clients include well-characterized CDPK substrates such as fructose-2,6-bisphosphatase (At1g07110), respiratory burst oxidase homologue D (At5g47910), syntaxin of plants 122 (At3g52400), DHHC-type zinc finger family protein (At3g48760), acetyl-CoA synthetase (At5g36880) nitrate reductase 2 (At1g37130), CRKs (A3g49370, At1g49580), calcium-binding EF-hand family proteins (At1g21630, At1g20760), aldehyde dehydrogenase 3 (At4g34240), and BRI1 suppressors 1 like proteins (At4g03080, At1g08420). Together with these proteins, a number of transporters, including PIP2.8 (At2g16850), MSL10 (At5g12080), Metal transport protein 5 (At3g12100), Transmembrane amino acid transporter family protein (At2g39130), and ATP-binding cassettes (At1g17840, At1g5987, At3g62700) were also identified as clients. It has also been reported that CPKs work as upstream signals for direct phosphorylation and the activation of many kinases including mitogen-activated protein kinase [74]. Consistent with this hypothesis and observation, we identified a number of kinases, such as CPK8 (At5g19450), Integrin-linked protein kinase family (At2g31800), CDC2a/CDK2 (At3g48750), ATMRK1 (At3g63260), LRR protein kinase family protein (At2g36570), MKP1 (At3g55270), and MKK1 (At4g26070), as potential downstream targets for CPKs. It is important to note that CPK1 was the only kinase that was common between the current study and the earlier peptide-based in vitro client hunting study performed Curran et al. [29]. However, CPK17 and CPK34, CPK28, and CPK16 are members of the same CDPK subfamily (Figure 1A). Moreover, few peptide substrates were in common between the studies. Therefore, a low number of overlapping clients between these two studies was not surprising.

### 3.5. Identification of Kinase-Specific Motifs

**Figure 4:**
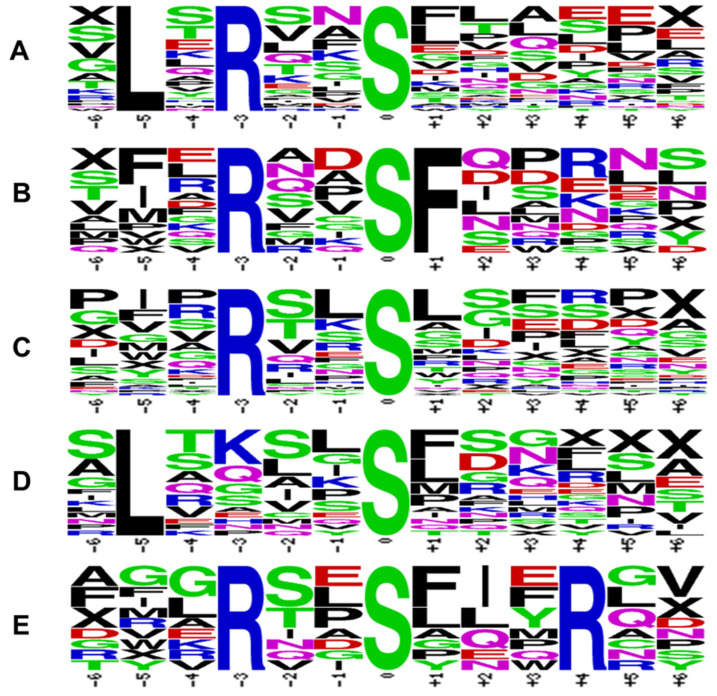
Amino acid sequences flanking the experimentally determined sites of peptide O phosphorylation. (**A**) .L.R..S…… (CPK3/17); (**B**) …R..SF….. (CPK17); (**C**) …R..S…… (CPK17/24); (**D**) .L….S…… (CPK17); (**E**) …R..S…R.. (CPKs). The CDPKs show preference for a basic residue (R/K) at positions −3/+4 and a hydrophobic residue (L/F) at positions −5/+1 to P-Ser (S). Sequence pileups were generated using the http://weblogo.berkeley.edu/logo.cgi algorithm (accessed on 14 April 2024).

Flanking amino acid residues around the phosphoacceptor sites play an essential role in kinase recognition, interaction, and the phosphorylation of the substrates. It has been proposed that members of the CPK family can recognize three distinct motifs: the classic motif/simple-1 motif [φ−5-x-R_−3_-x-x-S_0_-], ACA2 motif [R_−9_-R_−8_-x-R_−6_]-φ−5-x-x-x-x-S_0_-x-R_+2_], and ACS motif [φ−3-R−2-φ−1-S_0_-φ+1-x-K_+3_-R_+4_] (where φ is a hydrophobic residue) [75]. To elucidate the characteristic distribution of amino acid residues around the CPK-SnRK superfamily kinase phosphoacceptor sites, the resulting data set of phosphorylated clients/peptides was evaluated with Motif-X to extract over-represented patterns from the identified phosphopeptides and to determine the enrichment of specific amino acids at positions surrounding the phosphosite. The overall motif of the CDPK-SnRK superfamily kinases was detected by WebLogo, considering five high-stringency CPK phosphorylation site motifs are predominant in the mapped phosphosites (Figure 4). The motifs LxRxxS and xxxRxxS were in common with CPK3 and CPK24, respectively. The motifs xxxRxxSF and xLxxxxS were exclusive to CPK17, and the RxxSxxxR motif was common for all CPK clients. These data are in agreement with the known CPK motifs [29]. Additionally, the identification of new consensus sequences may provide valuable clues for CPK–client interaction and signaling pathways. Surprisingly, there were no significant motifs identified in this study for either the SnRK or PPCK kinase subfamilies. A total of four motifs were identified for CPK17 clients (Figure 4).

## 4. Discussion

We identified a broad set of CSS kinase clients using a peptide library of 2095 peptides derived from experimentally discovered, in vivo phosphorylation events in Arabidopsis. To estimate the kinase activity and avidity towards each phosphorylation site, we employed label-free spectral counting to monitor the stoichiometry of phosphorylation. We identified a total of 625 in vitro phosphorylation sites that could be assigned to 203 non-redundant client proteins. These sites consisted of 72% Ser, 23% Thr, and 5% Tyr, distributions that are comparable with reports from large-scale phosphoproteomic analyses in *O. sativa* and *A. thaliana*, wherein phosphorylation occurred at 85/83% Ser, 12/13% Thr, and 3/4% Tyr, respectively [76,77]. More than 1000 Tyr-phosphorylated proteins have been identified from proteomic analyses of plants [76,77,78]. Furthermore, a recent review summarized the current status of Tyr phosphorylation in plants indicating that the number is comparable to mammalians when various complementary methods and model systems are used [79]. Thus, the results of this study suggest that CPK-related PKs considerably contribute to plant Tyr phosphorylation (Figure 2). This represents an important distinction from mammalian systems where most of protein Tyr phosphorylation can be attributed to RK’s/RLK’s [80].

Among the identified 197 CPK clients, a significant number, 25 (13%), of previously established CPK clients were also detected here (Figure 3). For example, nitrate reductase 2, calmodulin-binding transcription activator 1, plasma membrane protein, CRK6, CRK8, calcium-binding EF-hand family protein, AtRbohD, and syntaxin were identified as clients for one or more CPKs (Figure 3). This result provides functional validation of the recombinant CSS kinases employed in this study. The subcellular localization of clients showed a good correlation with the CDPK responsible for their phosphorylation (Figure 3). Some of the known CPK targets were not detected by the KiC assay, most likely because the peptide substrate was not present in the assay. Additionally, undetected targets may have arisen due to several factor, such as (i) changes in the CPK target repertoire due to differences in experimental assay conditions, including for example differences in reported calcium concentration, pH, and assay duration; (ii) the effect of autoinhibitory autophosphorylation, influenced by assay conditions, on kinase target repertoire and activity; (iii) differences in kinase activity based on native vs. recombinant expression, the conditions of which can also influence the initial autophosphorylation status; and (iv) insufficient mass spectrometer sensitivity to detect low levels of phosphorylation occurring on some peptide targets. Among the analyzed CPKs, CPK17 had the largest number of specific clients, and CPK17 phosphopeptides/clients showed at least four significant motifs. Moreover, 60% of the CPK17 clients showed similar subcellular co-localization indicating some or all of these candidates are potentially authentic in vivo clients. Consistent with our findings, a large number of clients were established for CPK34 [29]. It is interesting to note that CPK17 and CPK34 are both categorized under the D subfamily (Figure 1A), suggesting that D subfamily members are more promiscuous than many of the other subfamilies of CPKs.

In this study, we provided a large number of clients from which interacting networks in response to calcium homeostasis could be deduced. For instance, 36 clients were identified for CPK1 that are targeted to peroxisome and lipid body membranes by N-myristoylation [81,82]. CPK1 is involved in salicylic acid accumulation, expressed by fungal elicitors, and is recognized to have a role during biotic and abiotic stresses [81,83]. The putative RING-H2 zinc finger protein toxicos en levadura 6 (ATL6) was defined herein as a CPK1 client. Similar to CPK1, ATL6 is induced upon elicitors, and *Pseudomonas syringae* treatment [84] and its overexpressor line demonstrated pathogen resistance [81,83]. Taken together, CPK1 could regulate ATL6 function through phosphorylation. Another identified CPK1/CPK3 client is defective kernel 1 (DEK1; At1g55350), which is a membrane protein of the calpain gene superfamily and is a Ca^2+^-dependent cysteine-type endopeptidase. DEK1 is required for aleurone cell development in maize grain endosperm and is involved in plant signal transduction [85]. Finally, CPK1 client *sensitivity to red light reduced 1* (*SRR1*) is upregulated by cytosolic Ca^2+^ changes [86] and is involved in the normal function of the normal oscillator during circadian rhythm and in phytochrome B (phyB)-mediated light signaling.

Kaplan et al. [87] identified 230 Ca^2+^ responsive genes, including 162 upregulated candidates, and identified the ABA-responsive element (ABRE) in their promoters [87]. Aconitase 3 (*Aco3*; At2g05710) expression is upregulated by cytosolic Ca^2+^ and contains ABRE in its promoter; it was found to be a client for both CPK1 and CPK17 in our screening. Aconitase 3 converts citrate to isocitrate and is a non-peroxisomal enzyme that is needed for the glyoxylate cycle, with involvement in oxidative stress and ABA response [88]. During germination, citrate is exported from the peroxisome to be converted to isocitrate. The fact that CPK1 is also associated with the outer surface of the peroxisome membrane could implicate it in regulating the supply of isocitrate to the peroxisome for glyoxylate cycle completion.

It is known that CPK3 and CPK6 regulate stomata closure as the double mutant was impaired in the ABA activation of the slow anion channel (SLAC) and Ca^2+^ permeable channels [89]. The aquaporin plasma membrane intrinsic protein (PIP2-8) was found to be upregulated upon Ca^2+^ treatment [86] and found to be a client for CPK17, CPK8, and CPK28 in this study, and its phosphorylation event was reported to be quantitatively altered upon ABA treatment [90]. Taken together, a regulatory function for Ca^2+^ signaling can be proposed through the phosphorylation of aquaporins through CPKs for regulating stomata openings as well as in response to abiotic stresses. Alcohol dehydrogenase 1 (ADH1) and pleiotropic drug resistance 8 (ABCG36), the stress-related proteins upregulated upon Ca^2+^ treatment [86], were defined as CPK17 clients. ABCG36 is a plasma membrane protein, and an ATP binding cassette transporter that contributes to SA-dependent biotic stress, and its phosphorylation status was found to be important during pathogen infection [91].

The zinc finger (C3HC4-type RING finger; At1g08050) family protein, which was downregulated upon cytosolic Ca^2+^ levels [86], was a client for PPCK1 and three SnRKs (1.3, 2.8, and 2.10). This protein was also found to be induced in early salicylate- and flg22-responsive redox-sensitive proteins in Arabidopsis [92]. At5g47430 (CCHC-type zinc finger) and At2g32450 (Calcium-binding tetratricopeptide family protein) are upregulated in response to Ca^2+^ and were clients for SnRK2.10. The function of these two genes is presently unknown. Based on our results, we hypothesize that SnRKs and PPCK1 might be phosphorylating and regulating a class of proteins, which indicates a pleiotropic way for the CSS members to manipulate stress response and signal transduction.

## Data Availability

The following Supporting Information is available free of charge at https://doi.org/10.5281/zenodo.10975938 (accessed on 14 April 2024).

## Supplementary Materials

The following supporting information can be downloaded at: https://www.mdpi.com/article/10.3390/plants13111481/s1 and https://doi.org/10.5281/zenodo.10975938 (accessed on 14 April 2024), Figure S1: Physiochemical Properties of the 2.1K (2095) synthetic peptides used in KiC assay for identification of potential CSS kinase clients. A, Group distribution of synthetic peptides based on hydrophobicity. B and C, Subcellular localization of the 2.1K peptide library corresponding proteins. C, Percentage of in vivo phosphosites of the 2.1K peptides. D, Distribution of five different hydrophobic groups of peptides in each peptide pool used in KiC assay; Figure S2: Diagrammatic representation of the KiC assay employed for CSS kinase client identification. Each pool was phosphorylated in vitro by individual CSS kinases and subsequently analyzed by an LTQ Orbitrap XL ETD mass spectrometer (Thermo Fisher, CA, USA). For identification and phosphosite localization, MS raw files were searched against a custom-made decoy database consisting of random complement of sequences comprising the peptide library, using SEQUEST (Proteome Discoverer, v. 1.0.3, Thermo Fisher); Figure S3: Kinase–client relationship between PPCKs and SnRKs. Venn diagram analysis showed the unique and overlapped clients between PPCKs (A) and among the SnRKs (B); Table S1: The list of the 2.1k synthetic peptides used for KiC assay; Table S2: List of the forward and reverse primers selected for cloning of protein kinases used in this study; Table S3: Putative CPK1 clients identified by KiC assay using a synthetic peptide library consisting of 2.1K peptides as substrates; Table S4: Putative CPK3 clients identified by KiC assay using a synthetic peptide library consisting of 2.1K peptides as substrates; Table S5: Putative CPK6 clients identified by KiC assay using a synthetic peptide library consisting of 2.1K peptides as substrates; Table S6: Putative CPK6 clients identified by KiC assay using a synthetic peptide library consisting of 2.1K peptides as substrates; Table S7: Putative CPK17 clients identified by KiC assay using a synthetic peptide library consisting of 2.1K peptides as substrates; Table S8: Putative CPK24 clients identified by KiC assay using a synthetic peptide library consisting of 2.1K peptides as substrates; Table S9: Putative CPK28 clients identified by KiC assay using a synthetic peptide library consisting of 2.1K peptides as substrates; Table S10: Putative CPK32 clients identified by KiC assay using a synthetic peptide library consisting of 2.1K peptides as substrates; Table S11: Putative PPCK1 clients identified by KiC assay using a synthetic peptide library consisting of 2.1K peptides as substrates; Table S12: Putative PPCK2 clients identified by KiC assay using a synthetic peptide library consisting of 2.1K peptides as substrates; Table S13: Putative SnRK 1.1 clients identified by KiC assay using a synthetic peptide library consisting of 2.1K peptides as substrates; Table S14: Putative SnRK 2.4 clients identified by KiC assay using a synthetic peptide library consisting of 2.1K peptides as substrates; Table S15: Putative SnRK 2.8 clients identified by KiC assay using a synthetic peptide library consisting of 2.1K peptides as substrates; Table S16: Putative SnRK 2.10 clients identified by KiC assay using a synthetic peptide library consisting of 2.1K peptides as substrates.

## References

[1] Balderas-Hernández V.E., Alvarado-Rodríguez M., Fraire-Velázquez S. (2013). Conserved versatile master regulators in signalling pathways in response to stress in plants. AoB Plants.

[2] Sheen J. (2010). Discover and connect cellular signaling. Plant Physiol..

[3] Van Norman J.M., Breakfield N.W., Benfey P.N. (2011). Intercellular communication during plant development. Plant Cell.

[4] Collaudin S., Mirabet V. (2014). Models to reconcile plant science and stochasticity. Front. Plant Sci..

[5] Murray J.A., Jones A., Godin C., Traas J. (2012). Systems analysis of shoot apical meristem growth and development: Integrating hormonal and mechanical signaling. Plant Cell.

[6] Popik O.V., Saik O.V., Petrovskiy E.D., Sommer B., Hofestädt R., Lavrik I.N., Ivanisenko V.A. (2014). Analysis of signaling networks distributed over intracellular compartments based on protein-protein interactions. BMC Genom..

[7] Plattner H., Verkhratsky A. (2015). The ancient roots of calcium signalling evolutionary tree. Cell Calcium.

[8] Spalding E.P., Harper J.F. (2011). The ins and outs of cellular Ca^2+^ transport. Curr. Opin. Plant Biol..

[9] Liese A., Romeis T. (2013). Biochemical regulation of in vivo function of plant calcium-dependent protein kinases (CDPK). Biochim. Biophys. Acta.

[10] Hepler P.K. (2005). Calcium: A central regulator of plant growth and development. Plant Cell.

[11] Galon Y., Finkler A., Fromm H. (2010). Calcium-regulated transcription in plants. Mol. Plant.

[12] Saidi Y., Finka A., Muriset M., Bromberg Z., Weiss Y.G., Maathuis F.J., Goloubinoff P. (2009). The heat shock response in moss plants is regulated by specific calcium-permeable channels in the plasma membrane. Plant Cell.

[13] Harada A., Shimazaki K.-I. (2009). Measurement of changes in cytosolic Ca^2+^ in *Arabidopsis* guard cells and mesophyll cells in response to blue light. Plant Cell Physiol..

[14] Toyota M., Furuichi T., Tatsumi H., Sokabe M. (2008). Cytoplasmic calcium increases in response to changes in the gravity vector in hypocotyls and petioles of *Arabidopsis* seedlings. Plant Physiol..

[15] Romeis T., Herde M. (2014). From local to global: CDPKs in systemic defense signaling upon microbial and herbivore attack. Curr. Opin. Plant Biol..

[16] Kiep V., Vadassery J., Lattke J., Maaß J., Boland W., Peiter E., Mithöfer A. (2015). Systemic cytosolic Ca^2+^ elevation is activated upon wounding and herbivory in *Arabidopsis*. New Phytol..

[17] Hamel L.-P., Sheen J., Séguin A. (2014). Ancient signals: Comparative genomics of green plant CDPKs. Trends Plant Sci..

[18] Harper J.F., Breton G., Harmon A. (2004). Decoding Ca^2+^ signals through plant protein kinases. Annu. Rev. Plant Biol..

[19] Lillo C., Kataya A.R.A., Heidari B., Creighton M.T., Nemie-Feyissa D., Ginbot Z., Jonassen E.M. (2014). Protein phosphatases PP2A, PP4 and PP6: Mediators and regulators in development and responses to environmental cues. Plant Cell Environ..

[20] Uhrig R.G., Labandera A.-M., Moorhead G.B. (2013). Arabidopsis PPP family of serine/threonine protein phosphatases: Many targets but few engines. Trends Plant Sci..

[21] Zulawski M., Schulze G., Braginets R., Hartmann S., Schulze W.X. (2014). The Arabidopsis Kinome: Phylogeny and evolutionary insights into functional diversification. BMC Genom..

[22] Nagata T., Iizumi S., Satoh K., Ooka H., Kawai J., Carninci P., Hayashizaki Y., Otomo Y., Murakami K., Matsubara K. (2004). Comparative analysis of plant and animal calcium signal transduction element using plant full-length cDNA data. Mol. Biol. Evol..

[23] Norris V., Grant S., Freestone P., Canvin J., Sheikh F.N., Toth I., Trinei M., Modha K., Norman R.I. (1996). Calcium signalling in bacteria. J. Bacteriol..

[24] Hrabak E.M., Chan C.W., Gribskov M., Harper J.F., Choi J.H., Halford N., Kudla J., Luan S., Nimmo H.G., Sussman M.R. (2003). The Arabidopsis CDPK-SnRK superfamily of protein kinases. Plant Physiol..

[25] Zou J.-J., Wei F.-J., Wang C., Wu J.-J., Ratnasekera D., Liu W.-X., Wu W.-H. (2010). Arabidopsis calcium-dependent protein kinase CPK10 functions in abscisic acid- and Ca^2+^-mediated stomatal regulation in response to drought stress. Plant Physiol..

[26] Cheng S.-H., Willmann M.R., Chen H.-C., Sheen J. (2002). Calcium signaling through protein kinases. The Arabidopsis calcium-dependent protein kinase gene family. Plant Physiol..

[27] Bender K.W., Blackburn R.K., Monaghan J., Derbyshire P., Menke F.L., Zipfel C., Goshe M.B., Zielinski R.E., Huber S.C. (2017). Autophosphorylation-based Calcium (Ca^2+^) Sensitivity Priming and Ca^2+^/Calmodulin Inhibition of *Arabidopsis thaliana* Ca^2+^-dependent Protein Kinase 28 (CPK28). J. Biol. Chem..

[28] Guo Y., Xiong L., Song C.-P., Gong D., Halfter U., Zhu J.-K. (2002). A calcium sensor and its interacting protein kinase are global regulators of abscisic acid signaling in *Arabidopsis*. Dev. Cell.

[29] Curran A., Chang I.-F., Chang C.-L., Garg S., Miguel R.M., Barron Y.D., Li Y., Romanowsky S., Cushman J.C., Gribskov M. (2011). Calcium-dependent protein kinases from *Arabidopsis* show substrate specificity differences in an analysis of 103 substrates. Front. Plant Sci..

[30] Matsoukas I.G. (2014). Interplay between sugar and hormone signaling pathways modulate floral signal transduction. Front. Genet..

[31] Umezawa T., Sugiyama N., Takahashi F., Anderson J.C., Ishihama Y., Peck S.C., Shinozaki K. (2013). Genetics and phosphoproteomics reveal a protein phosphorylation network in the abscisic acid signaling pathway in *Arabidopsis thaliana*. Sci. Signal..

[32] Zhang T., Chen S., Harmon A.C. (2014). Protein phosphorylation in stomatal movement. Plant Signal. Behav..

[33] Arsova B., Schulze W.X. (2012). Current status of the plant phosphorylation site database PhosPhAt and its use as a resource for molecular plant physiology. Front. Plant Sci..

[34] Wang P., Xue L., Batelli G., Lee S., Hou Y.-J., Van Oosten M.J., Zhang H., Tao W.A., Zhu J.-K. (2013). Quantitative phosphoproteomics identifies SnRK2 protein kinase substrates and reveals the effectors of abscisic acid action. Proc. Natl. Acad. Sci. USA.

[35] Yao Q., Ge H., Wu S., Zhang N., Chen W., Xu C., Gao J., Thelen J.J., Xu D. (2014). P^3^DB 3.0: From plant phosphorylation sites to protein networks. Nucleic Acids Res..

[36] Miernyk J.A., Randall D.D. (1989). A synthetic peptide-directed antibody as a probe of the phosphorylation site of pyruvate dehydrogenase. J. Biol. Chem..

[37] Ahsan N., Swatek K.N., Zhang J., Miernyk J.A., Xu D., Thelen J.J. (2012). “Scanning mutagenesis” of the amino acid sequences flanking phosphorylation site 1 of the mitochondrial pyruvate dehydrogenase complex. Front. Plant Sci..

[38] Gribskov M., Fana F., Harper J., Hope D.A., Harmon A.C., Smith D.W., Tax F.E., Zhang G. (2001). PlantsP: A functional genomics database for plant phosphorylation. Nucleic Acids Res..

[39] The Arabidopsis Genome Initiative (2000). Analysis of the genome sequence of the flowering plant *Arabidopsis thaliana*. Nature.

[40] Durek P., Schmidt R., Heazlewood J.L., Jones A., MacLean D., Nagel A., Kersten B., Schulze W.X. (2010). PhosPhAt: The *Arabidopsis thaliana* phosphorylation site database. An update. Nucleic Acids Res..

[41] Kemp B.E., Graves D.J., Benjamini E., Krebs E.G. (1977). Role of multiple basic residues in determining the substrate specificity of cyclic AMP-dependent protein kinase. J. Biol. Chem..

[42] Hashimoto Y., Soderling T.R. (1987). Calcium·calmodulin-dependent protein kinase II and calcium·phospholipid-dependent protein kinase activities in rat tissues assayed with a synthetic peptide. Arch. Biochem. Biophys..

[43] Wang Z. (2009). The peptide microarray-based assay for kinase functionality and inhibition study. Methods Mol. Biol..

[44] Xu J., Sun L., Ghosh I., Xu M.-Q., Kochinyan S., Barshevsky T. (2004). Western blot analysis of Src kinase assays using peptide substrates ligated to a carrier protein. Biotechniques.

[45] Fabian M.A., Biggs W.H., Treiber D.K., Atteridge C.E., Azimioara M.D., Benedetti M.G., Carter T.A., Ciceri P., Edeen P.T., Floyd M. (2005). A small molecule-kinase interaction map for clinical kinase inhibitors. Nat. Biotechnol..

[46] Kunz R.C., McAllister F.E., Rush J., Gygi S.P. (2012). A High-Throughput, Multiplexed Kinase Assay Using a Benchtop Orbitrap Mass Spectrometer To Investigate the Effect of Kinase Inhibitors on Kinase Signaling Pathways. Anal. Chem..

[47] Huang Y., Houston N.L., Tovar-Mendez A., Stevenson S.E., Miernyk J.A., Randall D.D., Thelen J.J. (2010). A quantitative mass spectrometry-based approach for identifying protein kinase clients and quantifying kinase activity. Anal. Biochem..

[48] Feilner T., Hultschig C., Lee J., Meyer S., Immink R.G.H., Koenig A., Possling A., Seitz H., Beveridge A., Scheel D. (2005). High throughput identification of potential *Arabidopsis* mitogen-activated protein kinases substrates. Mol. Cell. Proteom..

[49] Panse S., Dong L., Burian A., Carus R., Schutkowski M., Reimer U., Schneider-Mergener J. (2004). Profiling of generic anti-phosphopeptide antibodies and kinases with peptide microarrays using radioactive and fluorescence-based assays. Mol. Divers..

[50] Ptacek J., Devgan G., Michaud G., Zhu H., Zhu X., Fasolo J., Guo H., Jona G., Breitkreutz A., Sopko R. (2005). Global analysis of protein phosphorylation in yeast. Nature.

[51] Huang Y., Thelen J.J. (2012). KiC assay: A quantitative mass spectrometry-based approach for kinase client screening and activity analysis. Methods Mol. Biol..

[52] Ahsan N., Huang Y., Tovar-Mendez A., Swatek K.N., Zhang J., Miernyk J.A., Xu D., Thelen J.J. (2013). A versatile mass spectrometry-based method to both identify kinase client-relationships and characterize signaling network topology. J. Proteome Res..

[53] Olsen J.V., Blagoev B., Gnad F., Macek B., Kumar C., Mortensen P., Mann M. (2006). Global, in vivo, and site-specific phosphorylation dynamics in signaling networks. Cell.

[54] Zhu L., Li N. (2013). Quantitation, networking, and function of protein phosphorylation in plant cell. Front. Plant Sci..

[55] Brauer E.K., Ahsan N., Dale R., Kato N., Coluccio A.E., Piñeros M.A., Kochian L.V., Thelen J.J., Popescu S.C. (2016). The Raf-like Kinase ILK1 and the High Affinity K+ Transporter HAK5 Are Required for Innate Immunity and Abiotic Stress Response. Plant Physiol..

[56] Chen D., Cao Y., Li H., Kim D., Ahsan N., Thelen J., Stacey G. (2017). Extracellular ATP elicits DORN1-mediated RBOHD phosphorylation to regulate stomatal aperture. Nat. Commun..

[57] Li Z., Wang Y., Huang J., Ahsan N., Biener G., Paprocki J., Thelen J.J., Raicu V., Zhao D. (2017). Two SERK Receptor-Like Kinases Interact with EMS1 to Control Anther Cell Fate Determination. Plant Physiol..

[58] Chen D., Hao F., Mu H., Ahsan N., Thelen J.J., Stacey G. (2021). S-acylation of P2K1 mediates extracellular ATP-induced immune signaling in *Arabidopsis*. Nat. Commun..

[59] Brauer E.K., Ahsan N., Popescu G.V., Thelen J.J., Popescu S.C. (2022). Back From the Dead: The Atypical Kinase Activity of a Pseudokinase Regulator of Cation Fluxes During Inducible Immunity. Front. Plant Sci..

[60] Kim D., Chen D., Ahsan N., Jorge G.L., Thelen J.J., Stacey G. (2023). The Raf-like MAPKKK INTEGRIN-LINKED KINASE 5 regulates purinergic receptor-mediated innate immunity in *Arabidopsis*. Plant Cell.

[61] Bahk S., Ahsan N., An J., Kim S.H., Ramadany Z., Hong J.C., Thelen J.J., Chung W.S. (2024). Identification of mitogen-activated protein kinases substrates in *Arabidopsis* using kinase client assay. Plant Signal. Behav..

[62] Nozaki Y., Tanford C. (1971). The solubility of amino acids and two glycine peptides in aqueous ethanol and dioxane solutions. Establishment of a hydrophobicity scale. J. Biol. Chem..

[63] Bucholc M., Ciesielski A., Goch G., Anielska-Mazur A., Kulik A., Krzywińska E., Dobrowolska G. (2011). SNF1-related protein kinases 2 are negatively regulated by a plant-specific calcium sensor. J. Biol. Chem..

[64] McLoughlin F., Galvan-Ampudia C.S., Julkowska M.M., Caarls L., van der Does D., Laurière C., Munnik T., Haring M.A., Testerink C. (2012). The Snf1-related protein kinases SnRK2.4 and SnRK2.10 are involved in maintenance of root system architecture during salt stress. Plant J..

[65] Swaney D.L., McAlister G.C., Coon J.J. (2008). Decision tree–driven tandem mass spectrometry for shotgun proteomics. Nat. Methods.

[66] Schwartz D., Gygi S.P. (2005). An iterative statistical approach to the identification of protein phosphorylation motifs from large-scale data sets. Nat. Biotechnol..

[67] Rao R.S.P., Møller I.M. (2012). Large-scale analysis of phosphorylation site occupancy in eukaryotic proteins. Biochim. Biophys. Acta.

[68] Crooks G.E., Hon G., Chandonia J.-M., Brenner S.E. (2004). WebLogo: A sequence logo generator. Genome Res..

[69] Saito R., Smoot M.E., Ono K., Ruscheinski J., Wang P.-L., Lotia S., Pico A.R., Bader G.D., Ideker T. (2012). A travel guide to Cytoscape plugins. Nat. Methods.

[70] Furlotte N.A., Kang H.M., Ye C., Eskin E. (2011). Mixed-model coexpression: Calculating gene coexpression while accounting for expression heterogeneity. Bioinformatics.

[71] Uno Y., Rodriguez Milla M.A., Maher E., Cushman J.C. (2009). Identification of proteins that interact with catalytically active calcium-dependent protein kinases from *Arabidopsis*. Mol. Genet. Genomics.

[72] Vlad F., Turk B.E., Peynot P., Leung J., Merlot S. (2008). A versatile strategy to define the phosphorylation preferences of plant protein kinases and screen for putative substrates. Plant J..

[73] Mehlmer N., Wurzinger B., Stael S., Hofmann-Rodrigues D., Csaszar E., Pfister B., Bayer R., Teige M. (2010). The Ca^2+^-dependent protein kinase CPK3 is required for MAPK-independent salt-stress acclimation in *Arabidopsis*. Plant J..

[74] Xie K., Chen J., Wang Q., Yang Y. (2014). Direct phosphorylation and activation of a mitogen-activated protein kinase by a calcium-dependent protein kinase in rice. Plant Cell.

[75] Sebastià C.H., Hardin S.C., Clouse S.D., Kieber J.J., Huber S.C. (2004). Identification of a new motif for CDPK phosphorylation in vitro that suggests ACC synthase may be a CDPK substrate. Arch. Biochem. Biophys..

[76] Sugiyama N., Nakagami H., Mochida K., Daudi A., Tomita M., Shirasu K., Ishihama Y. (2008). Large-scale phosphorylation mapping reveals the extent of tyrosine phosphorylation in *Arabidopsis*. Mol. Syst. Biol..

[77] Nakagami H., Sugiyama N., Mochida K., Daudi A., Yoshida Y., Toyoda T., Tomita M., Ishihama Y., Shirasu K. (2010). Large-scale comparative phosphoproteomics identifies conserved phosphorylation sites in plants. Plant Physiol..

[78] Van Wijk K.J., Friso G., Walther D., Schulze W.X. (2014). Meta-Analysis of *Arabidopsis thaliana* Phospho-Proteomics Data Reveals Compartmentalization of Phosphorylation Motifs. Plant Cell.

[79] Ahsan N., Wilson R.S., Rao R.S.P., Salvato F., Sabila M., Ullah H., Miernyk J.A. (2020). Mass Spectrometry-Based Identification of Phospho-Tyr in Plant Proteomics. J. Proteome Res..

[80] Hunter T. (2014). The genesis of tyrosine phosphorylation. Cold Spring Harb. Perspect. Biol..

[81] Coca M., Segundo B.S. (2010). AtCPK1 calcium-dependent protein kinase mediates pathogen resistance in *Arabidopsis*. Plant J..

[82] Kataya A.R., Muench D.G., Moorhead G.B. (2019). A Framework to Investigate Peroxisomal Protein Phosphorylation in *Arabidopsis*. Trends Plant Sci..

[83] Huang K., Peng L., Liu Y., Yao R., Liu Z., Li X., Yang Y., Wang J. (2018). *Arabidopsis* calcium-dependent protein kinase AtCPK1 plays a positive role in salt/drought-stress response. Biochem. Biophys. Res. Commun..

[84] Hruz T., Laule O., Szabo G., Wessendorp F., Bleuler S., Oertle L., Widmayer P., Gruissem W., Zimmermann P. (2008). Genevestigator v3: A reference expression database for the meta-analysis of transcriptomes. Adv. Bioinform..

[85] Lid S.E., Gruis D., Jung R., Lorentzen J.A., Ananiev E., Chamberlin M., Niu X., Meeley R., Nichols S., Olsen O.-A. (2002). The *defective kernel 1* (*dek1*) gene required for aleurone cell development in the endosperm of maize grains encodes a membrane protein of the calpain gene superfamily. Proc. Natl. Acad. Sci. USA.

[86] Whalley H.J., Sargeant A.W., Steele J.F.C., Lacoere T., Lamb R., Saunders N.J., Knight H., Knight M.R. (2011). Transcriptomic analysis reveals calcium regulation of specific promoter motifs in *Arabidopsis*. Plant Cell.

[87] Kaplan B., Davydov O., Knight H., Galon Y., Knight M.R., Fluhr R., Fromm H. (2006). Rapid transcriptome changes induced by cytosolic Ca^2+^ transients reveal ABRE-related sequences as Ca^2+^-responsive *cis* elements in *Arabidopsis*. Plant Cell.

[88] Wang Y.-M., Yang Q., Liu Y.-J., Yang H.-L. (2016). Molecular Evolution and Expression Divergence of the Aconitase (ACO) Gene Family in Land Plants. Front. Plant Sci..

[89] Singh A., Sagar S., Biswas D.K. (2017). Calcium Dependent Protein Kinase, a Versatile Player in Plant Stress Management and Development. Crit. Rev. Plant Sci..

[90] Kline K.G., Barrett-Wilt G.A., Sussman M.R. (2010). In planta changes in protein phosphorylation induced by the plant hormone abscisic acid. Proc. Natl. Acad. Sci. USA.

[91] Underwood W., Somerville S.C. (2017). Phosphorylation is required for the pathogen defense function of the *Arabidopsis* PEN3 ABC transporter. Plant Signal. Behav..

[92] Liu P., Zhang H., Yu B., Xiong L., Xia Y. (2015). Proteomic identification of early salicylate- and flg22-responsive redox-sensitive proteins in *Arabidopsis*. Sci. Rep..

