# Supplementary material for: Decoding Arabidopsis thaliana CPK/SnRK Superfamily Kinase Client Signaling Networks Using Peptide Library and Mass Spectrometry
Source: Plants (Basel). 2024 May 27;13(11):1481. doi: 10.3390/plants13111481 (PMC11174488; doi:10.3390/plants13111481)
Supplement: Supplementary file 1 [file plants-13-01481-s001.zip › plants-2990542-supplementary.pptx]

## Slide 1
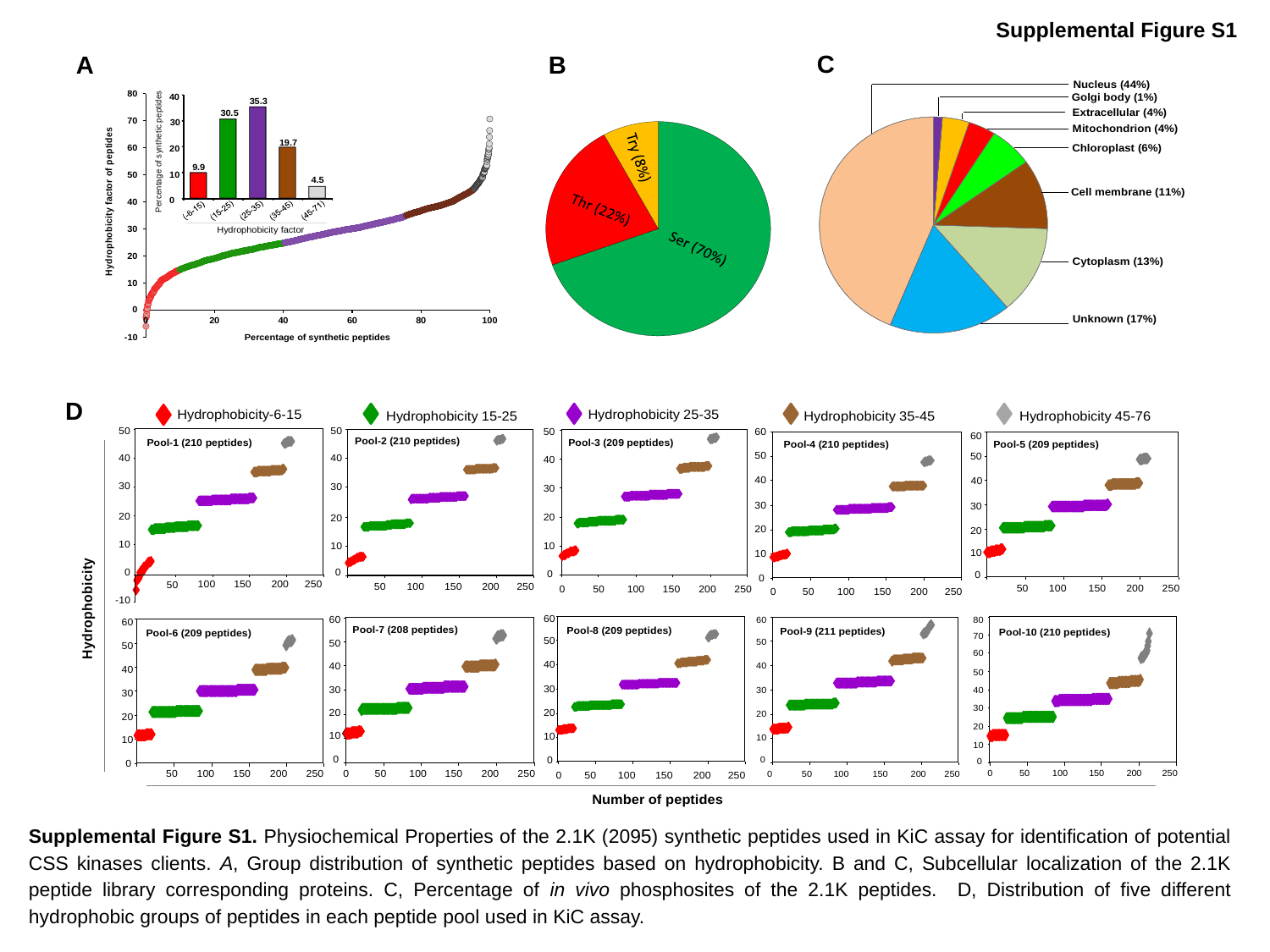

Supplemental Figure S1
C
A
B
D
Supplemental Figure S1. Physiochemical Properties of the 2.1K (2095) synthetic peptides used in KiC assay for identification of potential CSS kinases clients. A, Group distribution of synthetic peptides based on hydrophobicity. B and C, Subcellular localization of the 2.1K peptide library corresponding proteins. C, Percentage of in vivo phosphosites of the 2.1K peptides. D, Distribution of five different hydrophobic groups of peptides in each peptide pool used in KiC assay.

## Slide 2
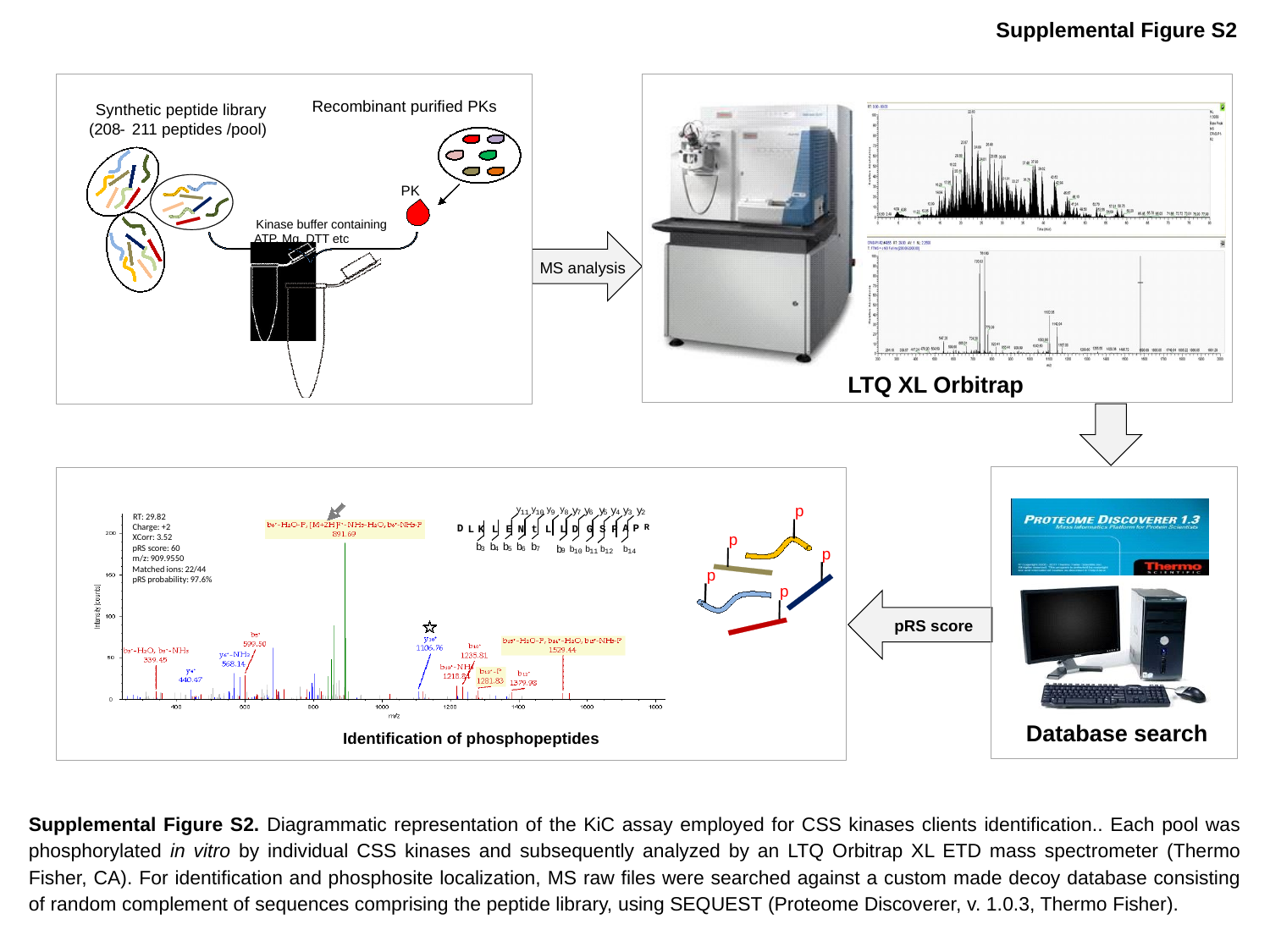

Supplemental Figure S2
Recombinant purified PKs
Synthetic peptide library
(208
-
211 peptides /pool)
PK
Kinase buffer containing
ATP, Mg, DTT etc
KiC Assay
MS analysis
LTQ XL Orbitrap
p
y
y
y
y
y
y
y
y
y
y
11
10
9
8
7
6
5
4
3
2
R
D
A
P
L
K
L
E
N
t
L
L
D
G
S
P
b
b
b
b
b
b
b
b
b
b
6
3
5
4
7
9
10
11
12
14
RT:
29.82
Charge: +2
XCorr
:
3.52
pRS
score:
60
m/z:
909.9550
Matched ions:
22/44
pRS
probability:
97.6%
p
p
p
p
pRS score
Database search
Identification of phosphopeptides
Supplemental Figure S2. Diagrammatic representation of the KiC assay employed for CSS kinases clients identification.. Each pool was phosphorylated in vitro by individual CSS kinases and subsequently analyzed by an LTQ Orbitrap XL ETD mass spectrometer (Thermo Fisher, CA). For identification and phosphosite localization, MS raw files were searched against a custom made decoy database consisting of random complement of sequences comprising the peptide library, using SEQUEST (Proteome Discoverer, v. 1.0.3, Thermo Fisher).

## Slide 3
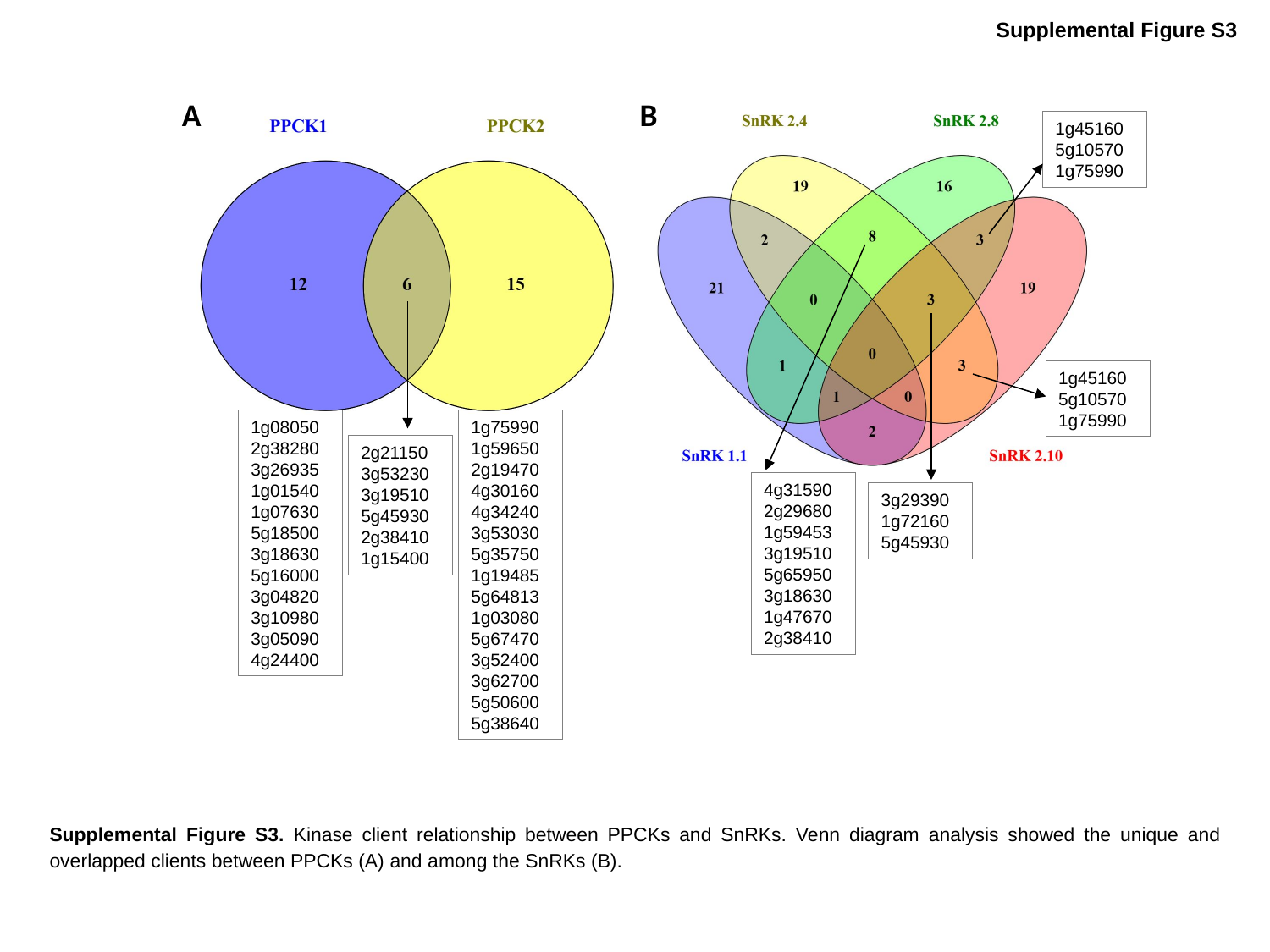

Supplemental Figure S3
A
B
1g08050
2g38280
3g26935
1g01540
1g07630
5g18500
3g18630
5g16000
3g04820
3g10980
3g05090
4g24400
1g75990
1g59650
2g19470
4g30160
4g34240
3g53030
5g35750
1g19485
5g64813
1g03080
5g67470
3g52400
3g62700
5g50600
5g38640
2g21150
3g53230
3g19510
5g45930
2g38410
1g15400
1g45160
5g10570
1g75990
1g45160
5g10570
1g75990
4g31590
2g29680
1g59453
3g19510
5g65950
3g18630
1g47670
2g38410
3g29390
1g72160
5g45930
Supplemental Figure S3. Kinase client relationship between PPCKs and SnRKs. Venn diagram analysis showed the unique and overlapped clients between PPCKs (A) and among the SnRKs (B).
